# Directional leads applied to spinal cord stimulation: A computational modelling study

**DOI:** 10.1371/journal.pone.0345287

**Published:** 2026-04-02

**Authors:** Zhen Wu, Nianshuang Wu, Penghao Wang, Cheng Zhang, Changzhe Wu, Xiaolin Huo, Guanghao Zhang

**Affiliations:** 1 Beijing Key Laboratory of Bioelectromagnetism, Institute of Electrical Engineering, Chinese Academy of Sciences, Beijing, China; 2 School of Electronic, Electrical and Communication Engineering, University of Chinese Academy of Sciences, Beijing, China; Coventry University, UNITED KINGDOM OF GREAT BRITAIN AND NORTHERN IRELAND

## Abstract

Spinal cord stimulation (SCS) is widely used to treat various types of pain. Directional leads, which exhibit directional focusing properties, have been applied in deep brain stimulation. Their targeting capability holds potential for further enhancing clinical SCS protocols. However, the actual stimulation effects of directional leads remain unclear and require further investigation. This study employed a computational modelling approach to develop SCS simulation models and a multi-compartment cable model of sensory fibers. These models were used to simulate the stimulation effects of directional leads in the human spinal cord. According to the reciprocity theorem, the evoked compound action potential (ECAP) generated by activated nerve fibers at recording contacts was calculated. Compared to traditional percutaneous leads, directional leads produced higher electric field strength and activating function in the spinal cord under the same current intensity, thereby activating a greater number of nerve fibers. Furthermore, increasing the angle between contacts further enhanced the advantages of directional leads. When the directional lead was rotated, the stimulation direction deviated from directly facing the spinal cord, resulting in a decreased number of activated nerve fibers. Under identical stimulation conditions, ECAP amplitudes recorded at different directional contacts showed variation; however, these differences remained within 6%. This study demonstrated the directional focusing advantage of directional leads and compared ECAP outcomes under varying conditions. The findings provide a solid theoretical foundation for the clinical application of directional leads.

## Introduction

Spinal cord stimulation (SCS), a neuromodulation technique used to treat various types of pain—including post-herpetic neuralgia and painful diabetic neuropathy—has been in clinical use for approximately six decades [1–3]. While SCS initially employed paresthesia to mask pain consistent with the “gate-control” theory proposed by Melczak and Wall in 1965 [4], modern SCS approaches use advanced waveforms to focus on the modulation of pain-relevant biochemical or molecular processes [5]. This theory suggests that activation of thin nerve fibers opens the “gate”, allowing pain signals to transmit to the central nervous system and generate pain perception, whereas activation of thick fibers closes the “gate”, thereby inhibiting the transmission of pain signals.

A typical SCS system consists of a pulse generator, electrodes, and connecting leads. By placing the electrode lead at a specific location within the epidural space of the spinal cord and delivering electrical pulses through the generator, the system modulates spinal cord activity to relieve pain.

SCS electrode leads are generally categorized into two types: percutaneous leads and paddle leads. Percutaneous leads consist of cylindrical, non-conductive polymer bodies with circular electrode contacts evenly spaced along the surface. Paddle leads, by contrast, feature circular or rectangular electrode contacts arranged on a flat surface. Although paddle leads are less prone to displacement and offer broader stimulation coverage, they require implantation via laminectomy, a procedure associated with greater surgical trauma and higher postoperative risk. In comparison, percutaneous leads are minimally invasive, safer, and more acceptable to patients [6]. However, they are more susceptible to displacement and typically contain a single rank of 8 contacts, resulting in reduced mediolateral coverage versus a paddle lead. To address complex pain conditions, dual-lead implantation has become a preferred strategy. Increasing the number of electrode contacts allows for more diverse stimulation configurations tailored to various clinical presentations [7]. By flexibly adjusting the position and number of cathodes and anodes according to clinical needs, dual-lead configurations improve pain coverage and mitigate the potential loss of efficacy due to lead displacement [8], thereby significantly enhancing the therapeutic flexibility of SCS. When the spacing between electrode contacts is minimized—that is, when cathode and anode contacts are placed closer together—the system can more precisely activate its primary target, the dorsal column (DC) fibers, while reducing the likelihood of activating dorsal root (DR) fibers [9]. Recently, a novel type of electrode lead, the directional lead, has been developed. Based on the structure of traditional percutaneous leads, the directional lead divides circular electrode contacts into three curved segments with different orientations. This design, already applied in deep brain stimulation to achieve directional stimulation, enables higher precision and selectivity in targeting specific neural regions [10]. The unique shape and arrangement of directional leads concentrate stimulation current in a particular direction, minimizing current dispersion. As a result, directional leads offer a promising approach for precise and individualized SCS treatment. However, their clinical efficacy remains uncertain and requires further investigation.

The evoked compound action potential (ECAP) generated by SCS is a physiological signal within the spinal cord that quantitatively reflects the extent of neural activation [11]. ECAP consists of action potentials from nerve fibers of various diameters and typically presents a triphasic waveform: an initial positive peak (P1), a sharp negative peak (N1), and a secondary positive peak (P2) [11,12]. The ECAP amplitude is defined as a positive voltage representing the voltage difference between the P2 peak and the N1 trough. As a quantitative indicator of nerve fiber activation, ECAP is also used as a control signal in closed-loop SCS systems. Clinical studies have shown that, compared to open-loop systems, closed-loop SCS system based on ECAP offers patients enhanced pain relief and more stable therapeutic outcomes [13,14].

Previous research has used computational models to simulate ECAP signals induced by SCS, enabling exploration of ECAP formation mechanisms and waveform-influencing factors [15–17]. Additionally, in vivo animal and human studies have recorded ECAP signals, confirming the validity of simulation-based results [18–21]. However, differences in ECAP signals recorded using directional leads versus traditional percutaneous leads have not yet been fully examined—an important gap that must be addressed to determine the clinical utility of directional leads.

In this study, we hypothesized that, compared to traditional percutaneous leads, directional leads could activate the same number of nerve fibers with lower stimulation current. Directional leads concentrate the stimulation current in a specific segmented direction, thereby avoiding the loss of current towards the ligament. To investigate the advantages of directional leads, we simulated and compared ECAP signals generated by directional and percutaneous leads using a two-stage modelling framework: a finite element model to simulate the SCS-induced electric field, and a multi-compartment cable model of sensory fibers. We analyzed differences in the range of neural activation and ECAP signals between the two lead types. Additionally, we evaluated how rotational changes and variations in electrode contact area of directional leads influence neural activation and ECAP waveform characteristics. The findings of this study provide strong theoretical support for the clinical implementation of directional leads.

## Methods

### Simulation model of SCS-induced electric field

**Fig 1:**
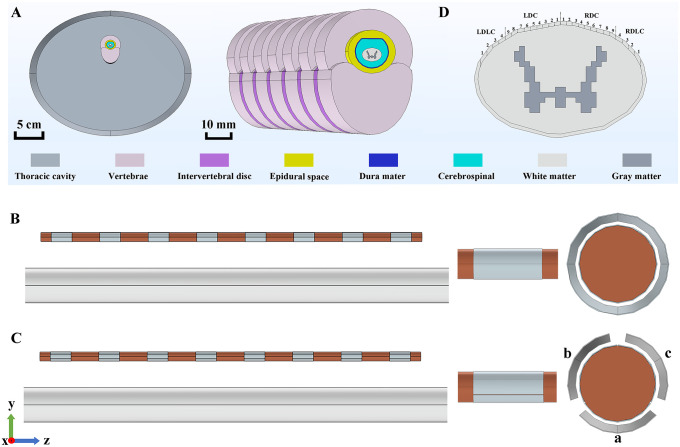
Simulation model of the human spinal cord and electrode leads. (A) The cross-sectional and left-front perspective views of the human spinal cord simulation model, including the overall structural diagram and an enlarged structural diagram after removing the thoracic cavity. (B) The model of the traditional percutaneous lead. (C) The model of the directional lead, where a, b, c represent three contact points in different directions (with the view on the far right being the top view along the z-axis). (D) The distribution of 26 regions within a 300 μm-thick layer on the outermost surface of the white matter. (Note: In the rightmost views of both (B) and (C), the electrode contact points have been magnified several times, and the angles between the contact points of the directional lead have also been amplified to ensure clarity in presentation. In reality, the thickness of the electrode contact points is only 20 μm, and all contact points are tightly adhered to the electrode lead. The angles between the contact points in different directions of the directional lead are only 2°.).

In this study, a simulation model of the human spinal cord was constructed using the finite element analysis software COMSOL Multiphysics 5.5 (COMSOL Inc., MA, USA). As shown in Fig 1A, the model comprises eight anatomical layers: gray matter, white matter, cerebrospinal fluid (CSF), dura mater, epidural space, vertebrae, intervertebral disc, and thoracic cavity. The geometric dimensions of each layer were defined according to values reported in the literature [22–25], with a CSF thickness of 3.2 mm and dura mater thickness of 300 μm [15]. The model includes seven vertebrae, all modelled based on the dimensions of the ninth thoracic vertebra (T9), with intervertebral discs connecting adjacent vertebrae. The conductivity values for each tissue are summarized in Table 1.

**Table 1:** Conductivity of various tissues.

Tissue	Conductivity (S/m)
Gray matter	0.230
White matter	(0.083, 0.083, 0.6)
Cerebrospinal fluid	1.700
Dura mater	0.600
Epidural space	0.250
Vertebrae	0.020
Intervertebral disc	0.650
Thoracic cavity	0.250
Scar tissue	0.110

Electrode leads were modelled in two configurations: a traditional percutaneous lead and a directional lead, as illustrated in Figs 1B and 1C, respectively. The traditional percutaneous lead measured 55 mm in length and 1.3 mm in diameter, featuring eight circular contacts evenly distributed along an insulated cylindrical body. Each contact was 3 mm in length with 4 mm inter-contact spacing [26]. A 300 μm layer of scar tissue surrounded the lead body [27]. The directional lead consisted of contacts subdivided into three circumferentially curved segments, each spanning an arc of 118°. The length and spacing of the contacts were identical to those of the traditional percutaneous lead, resulting in a total of 24 directional contacts.

All stimulation electrodes were configured in a bipolar arrangement and delivered monophasic stimulation. For the traditional percutaneous lead, the anode and cathode were assigned to contacts E6 and E7, respectively. According to literature [15], stimulation was delivered using ±4 mA at the respective contact surfaces, yielding a total current of 8 mA. The outer boundary of the thoracic cavity was set to ground. For the directional lead, contacts E6a (anode) and E7a (cathode), both directly facing the spinal cord, were used, also with a ± 4 mA stimulation current. To investigate the influence of contact orientation on neural activation, the directional lead was rotated by 30° and 60° in separate simulations. The Laplace equation was solved under steady-state conditions to obtain voltage distributions within the spinal cord.

### Multi-compartment cable model of sensory fibers

In this study, dorsal column (DC) fibers located within the outermost 300 μm of the white matter were categorized into nine types based on their diameters: 5.7 μm, 7.3 μm, 8.7 μm, 10.0 μm, 11.5 μm, 12.8 μm, 14.0 μm, 15.0 μm, and 16.0 μm [28]. The distribution of fiber diameters was obtained from morphometric analyses of human myelinated dorsal column fibers [29].

To streamline computational demands, the outer layer of the white matter was segmented into 26 equally spaced regions, including nine regions each for the left (LDC1–LDC9) and right (RDC1–RDC9) dorsal columns, as well as four regions each for the left and right dorsal lateral columns (LDLC1–LDLC4 and RDLC1–RDLC4), as depicted in Fig 1D. The distribution proportion of fibers with different diameters was set at 63.87%, 26.32, 7.37%, 1.75%, 0.35%, 0.19%, 0.08%, 0.04%, and 0.02% within the DC regions, and at 48.06%, 24.40%, 13.31%, 8.87%, 2.96%, 1.66%, 0.55%, 0.09%, and 0.09% within the DLC regions. The central axis of each region was designated as the trajectory of the representative fibers, and voltage distributions along these axes were extracted to serve as extracellular voltages for all fibers in the respective regions.

The sensory fiber model was based on the double-cable mammalian motor neuron model developed by McIntyre et al [29], later refined by Gaines et al [30] to represent mammalian sensory fibers. This model was implemented and simulated using NEURON (v8.2.6).

Given that morphological parameters such as node spacing and internodal length vary by fiber diameter, the extracellular voltage distributions were adjusted accordingly. These computations were performed using MATLAB R2017a (MathWorks, Natick, MA).

Voltage distributions derived from COMSOL simulations were subsequently applied to the sensory fiber model to analyze the activation characteristics of fibers of various diameters across different regions. Simulation parameters included a pulse width of 210 μs and a total simulation duration of 6 ms, with stimulation initiated at the 1 ms mark.

### Calculation of simulated ECAP signals

When a suprathreshold stimulus is applied to a nerve fiber, time-varying membrane currents are generated at the nodes of Ranvier, resulting in the formation of an action potential, also known as a single fiber action potential (SFAP). To simulate SFAPs, a 1 mA stimulus current (*i*_e_) was applied at the recording electrode contacts, with the outer surface of the thoracic cavity grounded. This stimulation generated an extracellular voltage (*V*_e_) at each node of Ranvier. Simultaneously, the membrane current (*i*_m_) at each node induced a corresponding voltage (*V*_m_) at the recording contacts. According to the reciprocity theorem [31], the relationship is given by:


$$V_{\text{m}}/i_{\text{m}}=V_{\text{e}}/i_{\text{e}}$$ (1)

Using this equation, the contribution of each node of Ranvier to the voltage *V*_m_ was calculated. Summing the contributions from all nodes yielded the time-varying SFAP waveform. Due to differences in conduction velocities across fiber diameters, the latency, amplitude, and waveform width of SFAPs vary accordingly. By aggregating the SFAPs of all fibers activated by SCS, the final simulated ECAP signal was obtained.

This process was repeated for different electrode contacts to generate monopolar ECAP signals at each site. By subtracting the signal of a designated reference contact from all others, bipolar differential ECAP signals were derived.

## Results

### Directional lead generates higher activating function

**Fig 2:**
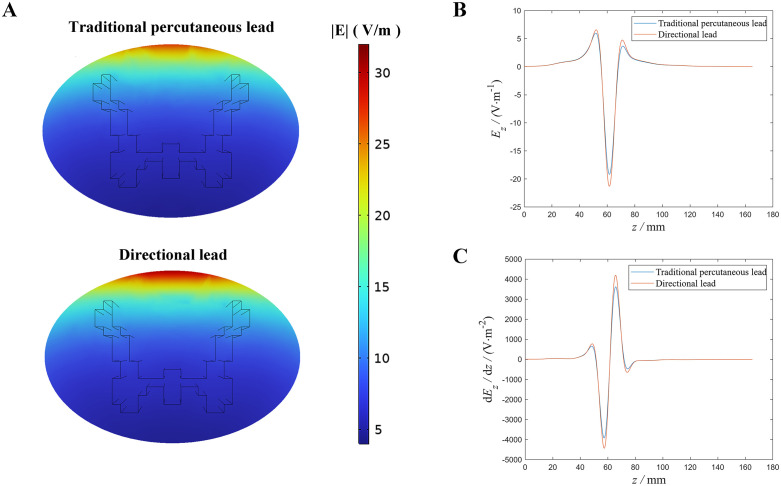
Distributions of electric field strength and gradient in the spinal cord generated by SCS at the current intensity of 8mA. (A) The cross-sectional distribution of the absolute values of the z-direction (axon direction) electric field components produced by two types of electrode leads within the white and gray matter. (B) The z-axis electric field component (*E*_z_) within the LDC1 region for both electrode leads. (C) The gradient of the z-axis electric field component (*E*_z_) within the LDC1 region for the two electrode leads.

As illustrated in Fig 2A, the directional lead produced a stronger electric field in the superficial layer of the white matter compared to the traditional percutaneous lead. Specifically, the maximum *E*_*z*_ component of the electric field in the LDC1 region reached −21.31 V/m with the directional lead, surpassing the −19.17 V/m generated by the traditional percutaneous lead, as shown in Fig 2B. Since the activating function (AF) is equal to the negative value of the electric field gradient [32], we calculated the *z*-axis electric field gradient (d*E*_*z*_/d*z*) in the LDC1 region for both leads, as presented in Fig 2C. The directional lead exhibited a maximum AF of 4433.14 V/m², notably higher than the 3921.07 V/m² recorded for the traditional percutaneous lead. These results suggest that the directional lead can generate a steeper AF, enhancing its effectiveness in activating dorsal column (DC) fibers.

### Directional lead activates larger fiber population

**Fig 3:**
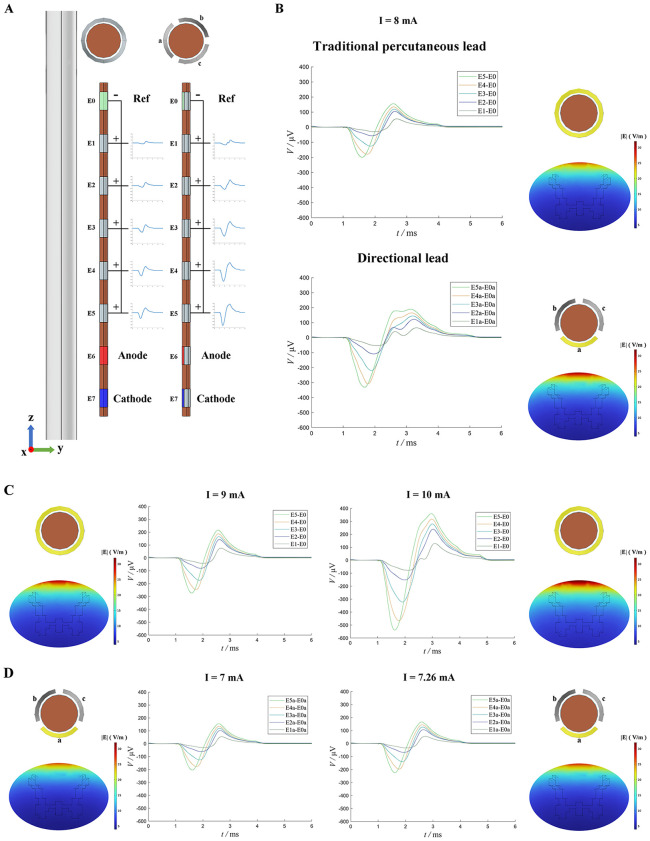
ECAP signals generated by two types of electrode leads under different current intensities. (A) Schematic diagram showing the relative positions of the electrode leads and white matter. E7 and E6 contacts were used as the cathode and anode of the stimulating contact pair, respectively, while E0 serves as the reference contact. The remaining contacts were recording contacts, and the bipolar ECAP signals were obtained by subtracting the reference contact signals. For the directional lead, the stimulating and recording contacts were both directly facing the spinal cord, specifically at contact a. (B) Bipolar ECAP signals obtained by the traditional percutaneous lead and directional lead under a current intensity of 8 mA. (C) ECAP signals obtained by the traditional percutaneous electrode at current intensities of 9 mA and 10 mA, respectively. (D) ECAP signals obtained by the directional lead at current intensities of 7 mA and 7.26 mA, respectively.

As shown in Fig 3B, at a stimulation current of 8 mA, the ECAP amplitude recorded between E3 and E0 (E3-E0) for the traditional percutaneous lead was 244.8 μV, whereas the E3a–E0a amplitude for the directional lead reached 363.6 μV—representing a 48.5% increase. Based on the calculation results, 5.68% of DC fibers were activated by the traditional percutaneous lead, while the directional lead activated a larger proportion, at 12.16%. The activation of smaller-diameter fibers by the directional lead also contributed to the appearance of a third positive peak in the ECAP waveform.

Maintaining the same electrode configuration, the current intensity for the traditional percutaneous lead was subsequently increased to 9 mA and 10 mA, resulting in E3–E0 amplitudes of 339.8 μV and 604.1 μV, respectively (Fig 3C). The corresponding fiber activation percentages were 7.94% and 18.66%. To compare the leads under similar activation conditions, a current of 7.26 mA was applied to the directional lead to match the AF generated by the traditional percutaneous lead at 8 mA. At this current, the directional lead yielded an ECAP amplitude of 266.2 μV with 6.26% fiber activation—still slightly higher than the 5.68% observed for the traditional percutaneous lead. Reducing the current to 7 mA resulted in an ECAP amplitude of 245.9 μV and fiber activation of 5.57%, closely aligning with the traditional percutaneous lead’s performance.

### The impact of the directionality of directional contacts

To evaluate the influence of contact orientation on fiber activation, the directional lead was rotated 30° and 60° clockwise while maintaining the same stimulation configuration and current intensity (8 mA). As shown in Table 2, the mean activation was high at 0°, and fell off with rotation. Specifically, the proportion of activated fibers decreased to 7.16% at 30° and 6.65% at 60° from 12.16% at 0°, indicating that deviation from optimal contact alignment (facing the spinal cord) led to reduced activation, with larger angles causing greater decreases.

**Table 2:** Mean activation at various rotation angles and corresponding ECAP amplitudes.

Rotation angle	Mean activation	E3a–E0a (μV)	E3b–E0b (μV)	E3c–E0c (μV)
0°	12.16%	363.6	348.0	348.0
30°	7.16%	313.9	297.5	304.6
60°	6.65%	285.8	273.3	284.4

**Fig 4:**
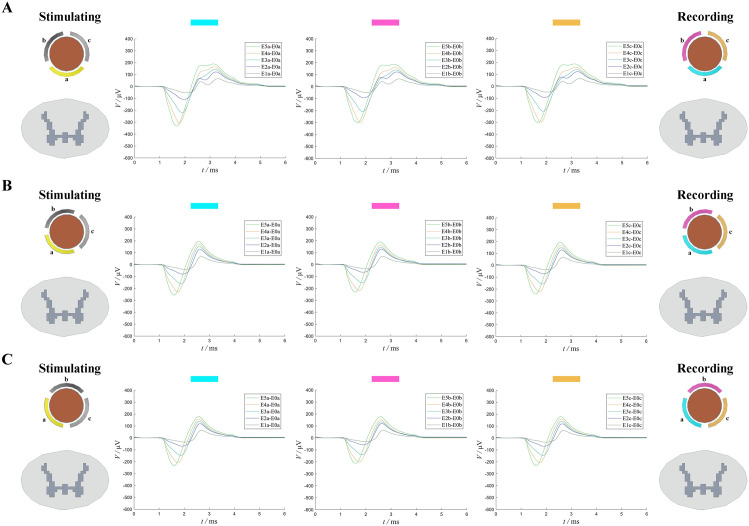
ECAP results with the directional lead rotated at different angles. (A) Results recorded by contacts a, b, and c when the directional lead was positioned with contact a directly facing the spinal cord (0°). (B) Results recorded by contacts a, b, and c when the directional lead was rotated to 30°. (C) Results recorded by contacts a, b, and c when the directional lead was rotated to 60°.

In contrast to the traditional percutaneous lead, the directional lead allows ECAP recording from any of three directional contacts (a, b, and c). Due to varying distances between these contacts and the spinal cord, recorded ECAP amplitudes differ. To assess this effect, three scenarios were compared, as shown in Fig 4 and Table 2. These findings demonstrate that while directionally distinct contacts produce slightly different ECAP recordings, the variations remain within a 6% margin.

### Effect of the contact angle of directional leads on fiber activation

**Fig 5:**
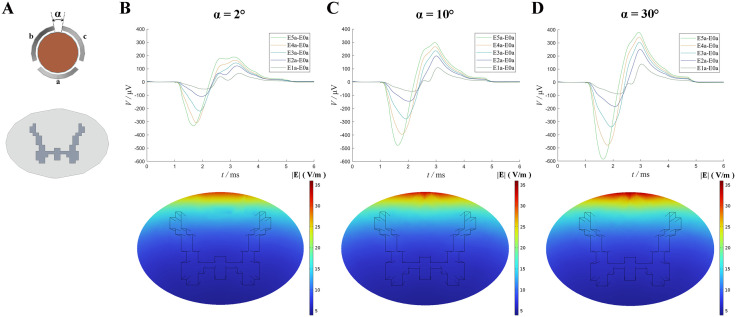
ECAP recorded under different contact angles. (A) Schematic illustration of contact angles. (B) ECAP results obtained with a contact angle set to 2°. (C) ECAP results obtained with a contact angle set to 10°. (D) ECAP results obtained with a contact angle set to 30°.

To further explore the effect of contact geometry, we varied the contact angle α of the directional lead from the default 2° to 10° and 30°, with the *a*-direction contact facing the spinal cord in all cases. Both stimulation and recording were conducted using the *a*-direction contact. As shown in Fig 5, increasing the contact angle enhanced activation: (i) At 10°, the ECAP amplitude reached 515.4 μV, with 14.96% fiber activation. (ii) At 30°, the amplitude increased to 644.5 μV, and activation reached 18.18%. These results indicate that a wider contact angle enhances fiber activation under the same stimulation intensity.

### The relationship between electric field strength, AF, and fiber activation

**Fig 6:**
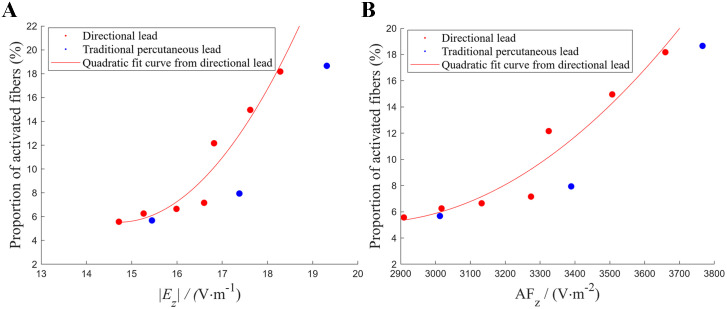
The relationship between electric field strength, AF, and fiber activation. (A) The relationship between the mean absolute value of the z-direction electric field component in 26 regions of the white matter surface and the proportion of activated fibers. (B) The relationship between the mean value of the z-direction AF in 26 regions of the white matter surface and the proportion of activated fibers. The scatter points represent the simulation results: red points correspond to results from the directional lead, and blue points correspond to results from the traditional percutaneous lead. The red curve is a quadratic fit curve derived from the results of the directional lead.

A statistical analysis of the average electric field strength, AF, and the corresponding fiber activation rates under various lead configurations is presented in Fig 6. A positive correlation was observed between fiber activation and both the electric field strength and AF, and the fitted curve for the directional lead was steeper. Interestingly, despite generating similar average field parameters, the traditional percutaneous lead activated fewer fibers. This may be attributed to its more dispersed current distribution, which reduces the local effectiveness of stimulation.

## Discussion

In this study, we developed a computational model to simulate SCS using directional leads and compared their performance to traditional percutaneous leads. Specifically, we analyzed the electric field distributions and nerve fiber activation patterns generated by both lead types under varying current intensities. Additionally, we investigated the impact of directional lead rotation and contact angle on stimulation outcomes, as well as the characteristics of ECAP signals recorded from differently oriented contacts under various conditions.

***To our knowledge, this is the first study on the electric field and the range of nerve fiber activation generated by the directional lead in SCS.*** Fiber activation is primarily determined by the AF along the axis of the nerve fibers [32]. Due to the directional focusing characteristics of directional leads, more current is concentrated through the small electrode contacts facing the spinal cord, resulting in higher local current densities. Our simulations demonstrated that at a stimulation current of 8 mA, the number of activated DC fibers with the directional lead was more than double that activated by the traditional percutaneous lead—highlighting the current-focusing advantage of directional designs.

When current intensities of 8 mA, 9 mA, and 10 mA were applied to the traditional percutaneous lead, the percentages of activated DC fibers were 5.68%, 7.94%, and 18.66%, respectively. In contrast, the directional lead activated 5.57%, 6.26%, and 12.16% of fibers at 7 mA, 7.26 mA, and 8 mA, respectively. Notably, at 7 mA, the directional lead achieved a similar activation level to that of the traditional percutaneous lead at 8 mA, indicating that directional leads can achieve equivalent therapeutic effects at lower stimulation intensities. Furthermore, the results showed that fiber activation increases nonlinearly with current intensity: at higher currents, finer and more densely distributed fibers begin to activate, leading to a rapid rise in the total number of activated fibers. However, as the current continues to increase, the growth rate slows, approaching a saturation point.

To further explore the influence of contact directionality, we simulated directional lead rotation angles of 30° and 60° (Fig 4). As the angle deviated from the optimal alignment, the percentage of activated fibers dropped from 12.16% to 7.16% and 6.65%, respectively. These findings confirm that improper alignment of stimulation contacts significantly reduces stimulation efficacy, consistent with previous studies [33]. Additionally, we examined the effect of increasing the angular spacing between contacts. The results showed that larger contact angles led to greater fiber activation, suggesting a positive correlation between contact angle and activation level.

***This is also the first simulation study on SCS induced ECAP signals recorded by directional leads.*** We compared ECAP recordings from both lead types at 8 mA (Fig 3B). Based on modelling literature [15], the E3-E0 configuration was used for comparison, as it more closely mirrors clinical recordings. At 8 mA, the E3-E0 amplitude from the traditional percutaneous lead was 244.8 μV, consistent with previous reports [15]. In contrast, the E3a-E0a amplitude recorded from the directional lead reached 363.6 μV—significantly higher than that of the traditional percutaneous lead. As current increased, ECAP amplitude also rose sharply, aligning with earlier findings [22].

Interestingly, although the directional lead activated slightly fewer fibers at 7 mA than the traditional percutaneous lead at 8 mA, it generated a higher ECAP amplitude. This suggests that the directional lead’s recording contacts may capture stronger ECAP signals. Moreover, the orientation of the recording contacts also affected the ECAP amplitude. We tested three rotational scenarios (0°, 30°, and 60°) and found that the ECAP amplitude differences across different contact orientations remained within 6% (Fig 4). From a clinical perspective, the influence of recording contact orientation on ECAP amplitude is minimal and can be regarded as negligible in practical applications.

***This study provided theoretical guidance for the clinical application of directional leads in SCS.*** Given the rapid increase in fiber activation with small increments in current, clinical systems should incorporate high-resolution current control to precisely modulate fiber recruitment and achieve more accurate pain management. Compared to traditional percutaneous leads, directional leads not only activate more fibers at the same current but also produce higher ECAP amplitudes. Therefore, ECAP-based programming strategies developed for traditional percutaneous leads may not be directly transferable and must be recalibrated for directional lead configurations.

Directional leads also present a novel solution to the challenge of electrode rotation in clinical practice. Because ECAP amplitudes vary among the three differently oriented contacts, and the contact closest to the spinal cord yields the highest amplitude, comparing ECAP signals from all three contacts can help infer the rotational orientation of the lead. By systematically simulating ECAP responses at various rotational angles, it may be possible to establish a quantitative relationship between lead rotation and ECAP asymmetry—offering a new method for assessing and correcting lead orientation.

Finally, increasing the angular separation between contacts was shown to improve fiber activation by enhancing local current density through smaller contact areas. However, in clinical practice, excessively small contact areas should be avoided, as they may lead to focal overstimulation, raising charge-safety concerns and potential tissue damage. Further work is required to delineate the optimal balance between angular separation and safety. Moreover, such excessive localized stimulation may inadvertently recruit Aδ or C fibers, potentially leading to unwanted side effects or discomfort.

### Limitations

This study also has several limitations. First, nerve fibers were constrained within a 300 μm-thick layer beneath the superficial surface of the dorsal column white matter and were divided into 26 regions. The extracellular voltage distribution for all fibers within each region was approximated using the voltage profile along the central axis of that region. This simplification fails to accurately reflect the spatial distribution of nerve fibers with varying diameters, as well as the local variations in extracellular potentials experienced by fibers at different depths and positions within the white matter. Furthermore, the stimulation targets were restricted to dorsal column fibers, omitting the recruitment of dorsal root fibers. This omission constrains the interpretation of selectivity and off-target activation. Future studies should incorporate anatomically accurate distributions of nerve fibers with varying diameters based on the actual organization of the human spinal cord, and extract region-specific extracellular voltage profiles to enable more realistic simulations of neural responses. Second, this study only investigated the stimulation effects of a bipolar configuration using a single directional lead, without exploring alternative configurations such as monopolar or tripolar arrangements, or multi-lead implants. A monopolar configuration would preferentially activate DR fibers, corresponding to a paresthesia coverage of one to two dermatomes [9]. In contrast, a tripolar configuration (guarded cathode) could achieve the maximal recruitment of DC fibers, thereby enabling a broader paresthesia coverage. Furthermore, multi-lead implants offer greater flexibility for therapeutic strategies. Future research should include a broader range of electrode configurations to better accommodate diverse clinical application scenarios and optimize stimulation strategies. Finally, challenges inherent to clinical SCS applications, such as CSF pulsation and spinal cord movement or lead displacement induced by postural changes, were not addressed in the current model. Future modeling efforts should prioritize investigating these critical issues to enhance the clinical relevance and predictive power of simulations.

## Conclusions

This study demonstrates that directional leads exhibit superior current-focusing capabilities compared to traditional percutaneous leads in SCS. Computational modeling showed that directional leads recruit a larger population of neural fibers under equivalent stimulation intensities, with this advantage becoming more pronounced as the inter-contact angle increases. Moreover, simulations of ECAP signals underscored the importance of contact orientation in shaping neural activation patterns, offering practical guidance for optimizing clinical SCS programming strategies. Together, these findings provide a theoretical framework for enhancing the precision and efficacy of directional lead applications in targeted neuromodulation therapies.
